# Structural compression and entorhinal vulnerability: Linking tentorial adjacency to tau burden and dementia progression

**DOI:** 10.1002/alz.71827

**Published:** 2026-10-05

**Authors:** Luyue Zhang, Ana M. Franceschi, John F. Crary, Frank A. Provenzano, for the Alzheimer's Disease Neuroimaging Initiative

**Affiliations:** ^1^ Department of Biomedical Engineering Columbia University New York New York USA; ^2^ Neuroradiology Division Department of Radiology Donald and Barbara Zucker School of Medicine at Hofstra/Northwell Lenox Hill Hospital New York New York USA; ^3^ Department of Pathology Nash Family Department of Neuroscience Department of Artificial Intelligence & Human Health Neuropathology Brain Bank & Research CoRE Ronald M. Loeb Center for Alzheimer's Disease Friedman Brain Institute Icahn School of Medicine at Mount Sinai New York New York USA; ^4^ Department of Neurology Columbia University Irving Medical Center New York New York USA

**Keywords:** aging, Alzheimer's disease, entorhinal cortex, mild traumatic brain injury, neuropathology, tau positron emission tomography, tauopathy

## Abstract

**INTRODUCTION:**

Early neurofibrillary tau degeneration in the entorhinal cortex (EC) is a hallmark of Alzheimer's disease (AD) and aging. We propose a biomechanical cascade hypothesis, suggesting proximity to the tentorial incisura (TI) may render the EC susceptible to chronic mechanical stress, potentially contributing to tau pathology.

**METHODS:**

We developed a neuroanatomical contact coefficient (NCC) to quantify EC–TI proximity using Alzheimer's Disease Neuroimaging Initiative multimodal imaging data (*n* = 47), stratifying participants into high and low adjacency groups.

**RESULTS:**

Controlling for risk factors, EC tau positron emission tomography signal predicted conversion from mild cognitive impairment to AD only in the high‐adjacency group (*P* = 0.036).

**DISCUSSION:**

Findings identify EC–TI proximity as a potential, novel, anatomically grounded biomarker of AD risk. These findings are consistent with a previously unrecognized biomechanical contribution to EC tau vulnerability in sporadic AD and aging, opening new avenues for early detection, risk stratification, and mechanistically targeted prevention strategies.

## Highlights

- A neuroanatomical contact coefficient (NCC) quantifies entorhinal–tentorial contact.
- Bilateral NCC stratifies risk for entorhinal tau accumulation in Alzheimer's Disease Neuroimaging Initiative.
- Entorhinal tau can only predict mild cognitive impairment to Alzheimer's disease conversion in a high‐risk group.
- Chronic biomechanical stress may trigger early entorhinal tau deposition.
- Entorhinal–tentorial adjacency links structural compression to dementia.

## 1 BACKGROUND

Deposition of neurofibrillary tangles (NFTs) composed of tau, a microtubule‐associated protein, is a hallmark of a group of disorders (“tauopathies”), with Alzheimer's disease (AD) being the most common.^1^, ^2^ In early AD, tau deposition is prominent in the entorhinal cortex (EC), a key structure within the hippocampal formation in the medial temporal lobe.^3^ Tauopathic changes are thought to progress over time, ultimately disrupting regional brain network dynamics.^4^

However, tau accumulation in the temporal lobe is not exclusive to AD. Primary age‐related tauopathy (PART) also features tau pathology confined predominantly to the medial temporal lobe.^5^ Unlike AD, PART lacks significant amyloid beta (Aβ) deposition and primarily affects elderly individuals, highlighting the complexity of tauopathies beyond amyloid‐related pathogenic processes.

The mechanism of the early selective vulnerability of the EC remains elusive. Activities on a cellular layer level may lead to degeneration, with no clear specific cell types appearing to drive neurodegeneration.^6^ The neurons in layer II of the EC are believed to be the initially vulnerable cells,^7^ with specific developmental, morphological, and biochemical features that may contribute to their vulnerability. Vascular factors, such as hypoperfusion, have also been proposed with some studies suggesting a negative correlation between blood flow and cognitive decline^8^ and as well as preceding tau deposition.^9^ Another hypothesis implicates high metabolic demands, as the EC functions as a major hub for integrating sensory information; its extensive connectivity may impose metabolic stress that heightens vulnerability.^10^ Inflammatory processes are also increasingly recognized as contributors to early pathology, possibly through microglial activation or cytokine‐mediated signaling cascades that exacerbate neuronal dysfunction.^11^ Genetic factors may modulate regional vulnerability through differential expression of proteins associated with neurodegeneration.^12^ Last, the accumulation of Aβ in and around the EC may compound these processes and contribute to disease onset.^13^

Tauopathy is also observed as a late event after mild yet repetitive traumatic brain injury (TBI). While TBI of varying severities shows acute symptomatologies, it can lead to long‐term, widespread complications.^14^ This pathological link connects TBI to an earlier onset of AD,^15^ an increased dementia risk by 2‐ to 4‐fold,^16^ and accelerated AD progression in animal models.^17^

The early accumulation of tau in TBI shares mechanistic similarities with chronic traumatic encephalopathy (CTE), in which repetitive mechanical trauma drives progressive tauopathy.^18^ Clinically, distinguishing between CTE and AD, particularly AD cases with a history of neurotrauma, can be challenging due to overlapping symptoms and pathological features.^16^ However, the spatiotemporal patterns of tau deposition differ between these conditions. In CTE, tau pathology spreads to the hippocampus and EC during stage III, whereas in AD, these regions are affected in the earliest stages.^19^

While external mechanical trauma, such as TBI, has been implicated in initiating tau propagation in AD,^20^ the role of intrinsic mechanical forces in tau accumulation remains largely unexplored. One potential mechanism involves chronic mechanical shearing forces from internal anatomical structures, potentially resulting in shearing forces and localized tau deposition. The EC is positioned adjacent to the rigid cerebellar tentorial wall, making it theoretically susceptible to mechanical compression (Figure 1A). However, there is a marked degree of interindividual variability in the size of the tentorial opening which would be predicted to modulate the degree of compression^21^ with the temporal lobe and middle cranial fossa likely depending on a larger cranial and cerebral system.^22^, ^23^

**FIGURE 1 alz71827-fig-0001:**
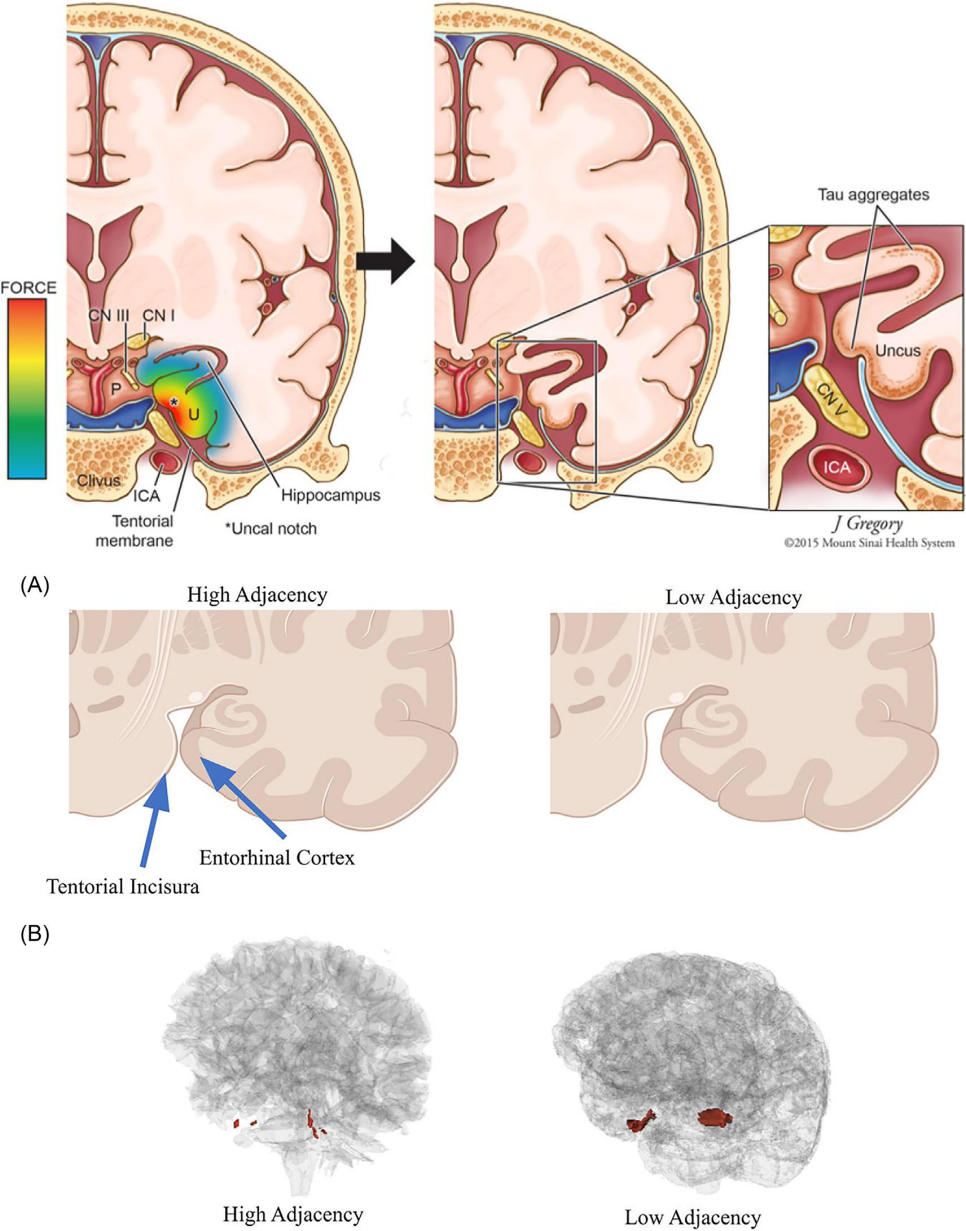
Entorhinal cortex is at risk of mechanical forces by tentorial incisura. The cohort is classified anatomically based on spatial proximity between the entorhinal cortex and tentorial incisura. Upper (a) shows the conceptual illustration of high‐risk (high anatomical adjacency; space proximity index < 500) (L) and low‐risk (low anatomical adjacency; space proximity index > 500) (R) configurations, and (b) the glass brains derived from two subjects’ channel space between the entorhinal cortex (EC) and tentorial incisura (TI). Red regions denote the segmented channel space.

To investigate this hypothesis, we used multimodal imaging including tau positron emission tomography (PET) and structural magnetic resonance imaging (MRI) in a cohort followed longitudinally. Our study sought to spatially quantify tau deposition in relation to anatomical sites of mechanical stress in the temporal lobe. Specifically, we examine how tau accumulation in EC can affect the conversion from mild cognitive impairment (MCI) to AD in patients with high and low entorhinal–tentorial (EC–TI) proximity defined in MRI, and test whether tau accumulation correlates with impingement against the TI. These findings could offer novel insights into the potential role of chronic mechanical forces in AD pathogenesis.

**RESEARCH IN CONTEXT**

1. **Systematic review**: The authors reviewed the literature using traditional online sources such as PubMed and meeting abstracts. While the chronic biomechanical stress entorhinal cortex endured that is relevant to Alzheimer's disease (AD) etiology is not as widely studied as more acute injuries (e.g., traumatic brain injury), there have been several publications describing the possible structural vulnerability and inherent structural protection aspects of brain regions relevant to neurodegeneration or other physiological systems. These relevant citations are appropriately referenced.
2. **Interpretation**: Our findings led to a hypothesis that suggests chronic direct compression from tentorial incisura to entorhinal cortex as a potential etiological contributor to the tau pathology in the initiation of AD. This hypothesis is consistent and contributes more detailed explanation with non‐clinical and clinical findings in the public domain at present.
3. **Future directions**: This article proposes a biomechanical cascade hypothesis for tau pathology initiating AD, and a biometric to evaluate the structural compression of the entorhinal cortex. It introduces further investigation to the structural mechanical properties of entorhinal–tentorium interface. Examples of future research include: (a) using finite element analysis to quantify shear stress and strain experienced at the tentorial notch (on entorhinal cortex), (b) assessing whether this anatomical risk factor should be used as a covariate to stratify patients in clinical trials for tau‐related therapies, and (c) investigating the relationships between biomechanical stress and genetic risk factors of AD onset.

## 2 METHODS

### 2.1 Subject admission and clinical data assessments

All participants were drawn from the Alzheimer's Disease Neuroimaging Initiative (ADNI) database (adni.loni.usc.edu),^24^ which offers open source neuroimaging and clinical data from elderly individuals at risk for AD. We identified and selected 47 participants with a baseline diagnosis of MCI, whose MRI and PET scans met ADNI quality control standards. Inclusion criteria required availability of T1‐weighted structural MRI, tau PET scans (University of California [UC] Berkeley—tau PET 6 mm res), and amyloid PET‐based status (UC Berkeley—amyloid PET 6 mm res), all collected within 12 months of baseline MRI acquisition. Of all individuals, 24 converted to AD within[Fig alz71827-fig-0001] 36 months of initial MCI diagnosis, while 23 remained stable. Demographic data, including age, sex, and Mini‐Mental State Examination (MMSE) scores, were acquired at baseline (Table 1).

**TABLE 1:** Demographic data and clinical assessment of participants that did or did not convert from MCI to AD (*n* = 47).

	Converted to AD within 36 months (*n* = 24)		Did not convert to AD within 36 months (*n* = 23)			Total	
	*n*/mean	%/SD	*n*/mean	%/SD	*p* value	*n*/mean	%/SD
Baseline age, years	74.13	7.71	74.30	7.61	0.941	74.21	7.58
Female, *n*	12	50.00%	9	39.13%	0.649	21	44.68%
MMSE	26.46	2.34	26.74	2.22	0.675	26.60	2.26
Amyloid positive, *n*	20	83.33%	13	56.52%	0.090	33	70.21%

Abbreviations: AD, Alzheimer's disease; MCI, mild cognitive impairment; MMSE, Mini‐Mental State Examination; SD, standard deviation.

### 2.2 MRI data acquisition and preprocessing

Structural MRI scans were obtained using the ADNI3 and ADNI4 protocol with 3 Tesla scanners and a magnetization‐prepared rapid acquisition gradient echo (MPRAGE) sequence (repetition time = 2300 ms; echo time = minimum full echo; inversion time = 900 ms; field of view = 208 × 240 × 256 mm; voxel size = 1 mm^3^). Preprocessing was conducted using FreeSurfer 6.0 and 7.3, depending on ADNI epoch,^25^ including skull stripping, intensity normalization, and segmentation of EC and hippocampus.^26^, ^27^ Total intracranial volume (TIV), EC, and hippocampus segmentations were extracted from the aparc+aseg.nii files by FreeSurfer.

### 2.3 EC–TI channel segmentation

To quantify anatomical proximity between the EC and the TI, we developed a semi‐automated segmentation pipeline combining FreeSurfer‐based anatomical priors with seed‐based expansion and manual refinement. EC binary masks were first generated using FreeSurfer's cortical parcellation, and served as anatomical references for identifying the channel space between the EC and TI. The segmentation workflow comprised four stages: medial cluster identification, low‐intensity voxel thresholding, seed‐based voxel expansion, and guided manual correction. This approach was designed to reduce the influence of noise and segmentation artifacts while preserving anatomical validity across participants.

To initialize the segmentation, we first isolated the two largest contiguous clusters from each hemisphere of the EC mask. These clusters provided stable EC masks by minimizing contributions from fragmented or spurious voxels, especially along the mediolateral boundary of EC. To define the medial portion of the EC in each coronal slice, we identified the most inferior voxel of the EC mask within that slice. A vertical line along the superior–inferior axis was then drawn through this point. EC voxels located medial to this vertical line were selected to form the medial EC mask. The medial EC mask served as a reference to find the starting seed for space searching within the channel between the EC and the TI.

Low‐intensity voxels corresponding to the air‐filled channel were identified using an adaptive thresholding strategy. Specifically, the mode intensity of EC voxels was computed, and a threshold was defined at 70% of this value to suppress signal contributions from surrounding gray matter and bone. Voxels within a 2‐voxel medial range of the medial EC mask and falling below this threshold were designated as seeds. An 8‐connected component expansion was then applied to every coronal slice. Each seed voxel was iteratively expanded to its 8‐connected neighbors if they satisfied both intensity and spatial constraints. This process produced a preliminary delineation of the channel between the EC and the TI.

After automatic segmentation, masks were manually refined to ensure anatomical accuracy. Refinement was guided by native‐space T1‐weighted MRI. Randomly selected masks were redone in a blinded manner to assess intra‐subject reliability (Dice index 0.967 ± 0.0099; Table S1 in supporting information). Skull‐stripped T1‐weighted MRI can also serve the purpose. Boundaries were adjusted using three constraints. First, channel masks were restricted within the outer boundary of the skull, with air‐filled pockets in the skull excluded. Second, masks were constrained between the lateral boundary of the skull and the medial boundary of the EC. Last, voxels within the rhinal sulcus (also known as the olfactory sulcus) were excluded to avoid false‐positive labeling. The resulting segmentations provided subject‐specific representations of the EC–TI channel suitable for morphometric analysis.

### 2.4 Neuroanatomical contact coefficient calculation and anatomical risk classification

Individual variation in the EC–TI channel spatial adjacency was quantified using a neuroanatomical contact coefficient (NCC) derived from segmented channel masks and absolute entorhinal volume. For each participant, left‐to‐right distances between the EC and TI were computed on each coronal slice where both structures were present. These distances were then summed across the full rostrocaudal extent to generate an absolute channel space NCC.

To adjust for differences in overall brain size, the absolute NCC was normalized by the EC volume raised to the ⅔ power, scaling the metric in surface area units. This normalization preserved morphometric interpretability while controlling for individual variability. For finer classification, the resulting proximity values were scaled by a factor of 100. The final output, termed the NCC, served as a continuous measure of EC–TI adjacency.

The NCC is calculated as follows:

$$\text{NCC}=100\times \left(\frac{\text{channel space volume}}{\text{EC}\;{\text{volume}}^{2/3}}\right)$$

Based on NCC, participants were stratified into anatomical risk groups. Hemispheric indices > 500 were labeled hemispheric low risk, while values ≤ 500 were classified as hemispheric high risk. Individuals were considered high risk overall only if both hemispheres met the high‐risk criterion, while all others were categorized as low risk. This binary classification was used in subsequent statistical models to test whether EC–TI proximity modulated the effects of tau pathology, regional atrophy, or conversion to AD.

### 2.5 Relative EC and hippocampus volume calculation

To account for individual variance, relative volumes were calculated by dividing each by the TIV, resulting in the EC–TIV and the hippocampus–TIV (Hippo–TIV) ratios. Regional volumes were extracted from masks generated by FreeSurfer, and TIV was derived as the sum of all volumes within the aparc+aseg.nii file. Hemispheric relative volumes were scaled by a factor of 2 × 10^5^, while the total relative volumes were scaled by a factor of 10^5^ for better distinction.

### 2.6 Statistical analysis

Statistical analyses were conducted in Python 3.0 using libraries including NumPy, Pandas, and Matplotlib. Two‐sample *t* tests were applied to normally distributed continuous variables (age, MMSE score, EC–TIV ratio, and tau PET standardized uptake value ratio [SUVR]). Chi‐squared tests were used for comparison in binary variables (sex, PET amyloid status, conversion, and EC anatomical risk). Statistical significance was set at *P* < 0.05. Pearson correlation was used to examine relationships among continuous variables, including relative EC and hippocampus volumes, tau PET SUVR, and the NCC. Multivariate logistic regressions were conducted within risk groups, with variable combinations to identify predictors of MCI‐to‐AD conversion. Significance was determined at *P* < 0.05 for individual predictors and at *P* < 0.05 for the model's likelihood‐ratio test (LLR *P*  value). No correction for multiple testing was applied given the exploratory nature of this analysis and *P*  values should be interpreted accordingly.

## 3 RESULTS

### 3.1 Baseline clinical and demographic characteristics of conversion groups

In summary of the baseline demographic and clinical characteristics of participants, we tested the data grouped by two criteria: (1) whether they converted from MCI to AD within 36 months of initial diagnosis and (2) the EC anatomical risk classification carried out in our study.

Among the 47 participants, 24 (51%) progressed to AD within 36 months, while 23 did not (Table 1). Baseline age (74.13 vs. 74.30 years; *P* = 0.94), sex distribution (50.0% vs. 58.3% female; *P* = 0.65), and MMSE scores did not differ between converters and non‐converters.

### 3.2 Baseline clinical and demographic characteristics of the EC anatomical risk groups

To further characterize our EC‐based anatomical risk classification, we compared demographic features across high‐ and low‐risk groups defined by spatial proximity indices developed for this study. The NCC is calculated in each hemisphere for every subject to quantify anatomical variation in the spatial relationship between the EC and the TI. This metric was derived by summing the mediolateral distance between EC and TI measured in coronal slices (specifically along the horizontal axis of the coronal scan), then normalizing by EC volume to the ⅔ power to account for differences in cortical surface area. The normalized NCC was scaled by 100 and used to assign lateral anatomical risk. Participants with indices < 500 in both hemispheres were classified as high risk; otherwise, they were assigned to the low‐risk group. Normally, with higher risk, the participant is more likely to have EC close to or abutting TI (Figure 1A,B). The distribution of relevant demographic and imaging metrics is shown in Figures S1–S2 in supporting information.

As expected, the left and right proximity indices were significantly (Table 2) higher in the high‐risk group (*P* = 0.0002 and 0.0001, respectively), which, because this grouping was determined by this very measure, serves as an internal check of methodological stability rather than an independent finding. Despite anatomical differences, the high‐ and low‐risk groups did not differ in baseline age (73.45 vs. 75.12 years, *P* = 0.48), sex (58.3% vs. 30.4% female, *P* = 0.103), MMSE score (26.83 vs. 26.35, *P* = 0.47), or amyloid positivity (66.7% vs. 73.9%). More importantly, the proportion of participants who converted to AD also showed no difference between groups (54.2% vs. 47.8%, *P* = 0.89). In conclusion, while proximity‐based risk groups reflect anatomical variation, they do not show distinction with clinical features or conversion outcomes.

**TABLE 2:** Demographic data, clinical assessment, left and right space proximity index, and conversion rates of participants (*n* = 47).

	High space risk (*n* = 24)		Low space risk (*n* = 23)		*p* value	Total	
	*n*/mean	%/SD	*n*/mean	%/SD		*n*/mean	%/SD
Baseline age, years	73.45	8.04	75.12	7.16	0.483	74.21	7.58
Female, *n*	14	58.33%	7	30.43%	0.886	21	44.68%
MMSE	26.83	1.83	26.35	2.66	0.468	26.60	2.26
Amyloid positive, *n*	16	66.67%	17	73.91%	0.823	33	70.21%
Left space proximity index	44.90	30.04	181.30	164.20	<0.0005	111.65	134.52
Right space proximity index	32.98	24.42	119.67	98.56	<0.0005	75.40	82.84
Conversion, *n*	13	54.17%	11	47.83%	0.886	24	51.06%

Abbreviations: AD, Alzheimer's disease; MCI, mild cognitive impairment; MMSE, Mini‐Mental State Examination; SD, standard deviation.

### 3.3 Space risk affects prediction of conversion to AD by tau PET SUVR

Focusing on the EC anatomical stratification, we further evaluated its biological relevance; specifically, we compared the relative EC and hippocampus (HC) volumes, regional tau PET SUVR, between high‐ and low‐risk groups defined by bilateral spatial proximity indices. Among lateral and overall EC and HC volumes, only the left HC showed a significant difference (Table 3). Subjects in the high‐risk group had larger left HC volumes compared to those in the low‐risk group (694.05 ± 98.40 vs. 633.93 ± 104.93; *P* = 0.049). Similarly, tau PET SUVR in both the EC and HC on the left and right side did not differ significantly between risk groups. A separate reduced parameter analysis including FreeSurfer version does not change the significance of the model (Table S2 in supporting information).

**TABLE 3:** Imaging metrics comparison between high channel risk group and low channel risk group.

	High channel space risk group (*n* = 24)				Low channel space risk group (*n* = 23)				
	Mean	SD	L95%CI	U95%CI	Mean	SD	L95%CI	U95%CI	*p* value
**EC size**									
EC/TIV ratio (x 10^5^)	294.36	50.54	273.58	315.14	288.20	61.34	262.44	313.96	0.709
LEC/TIV ratio (x 2 × 10^5^)	306.68	62.89	280.83	332.54	290.24	62.32	264.06	316.41	0.373
REC/TIV ratio (x 2 × 10^5^)	282.03	50.81	261.14	302.92	286.17	78.87	253.04	319.29	0.831
**HC size**									
HC/TIV ratio (x 10^5^)	687.18	88.60	650.75	723.60	648.04	69.15	619.00	677.08	0.099
LHC/TIV ratio (x 2 × 10^5^)	694.05	98.40	653.60	734.51	633.93	104.93	589.87	678.00	0.049
RHC/TIV ratio (x 2 × 10^5^)	682.35	111.38	636.56	728.14	672.24	103.26	628.87	715.61	0.749
**Tau SUVR**									
Tau PET SUVR in EC	1.440	0.301	1.316	1.563	1.474	0.302	1.347	1.601	0.700
Tau PET SUVR in HC	1.348	0.221	1.258	1.439	1.393	0.214	1.303	1.483	0.486

Abbreviations: EC, entorhinal cortex; HC, hippocampus; L95%CI, lower 95% confidence interval; LEC, left entorhinal cortex; LHC, left hippocampus; PET, positron emission tomography; REC, right entorhinal cortex; RHC, right hippocampus; SD, standard deviation; SUVR, standardized uptake value ratio; TIV, total intracranial volume; U95%CI, upper 95% confidence interval.

We examined whether regional brain volume was associated with tauopathy in the EC and HC. No significant correlation was found between relative EC volume (EC–TIV ratio) and EC tau SUVR (Figure 2A, 2B, 2C) in the full cohort (*r* = −0.18, *P* = 0.215), within the high‐ (*r* = −0.19, *P* = 0.379) or low‐risk groups (*r* = −0.18, *P* = 0.417). In contrast, a weak but significant negative correlation emerged between relative HC volume (HC/TIV ratio) and hippocampal tau SUVR (Figure 2D–F) in the full cohort (*r* = −0.33, *P* = 0.023). This relationship was not significant within the high‐risk (*r* = −0.34, *P* = 0.103) or low‐risk groups (*r* = −0.29, *P* = 0.183). Correlations among lateral NCC, lateral EC tau SUVR, relative EC volume, and EC cortical thickness are shown in Figures S3–S7 in supporting information.

**FIGURE 2:**
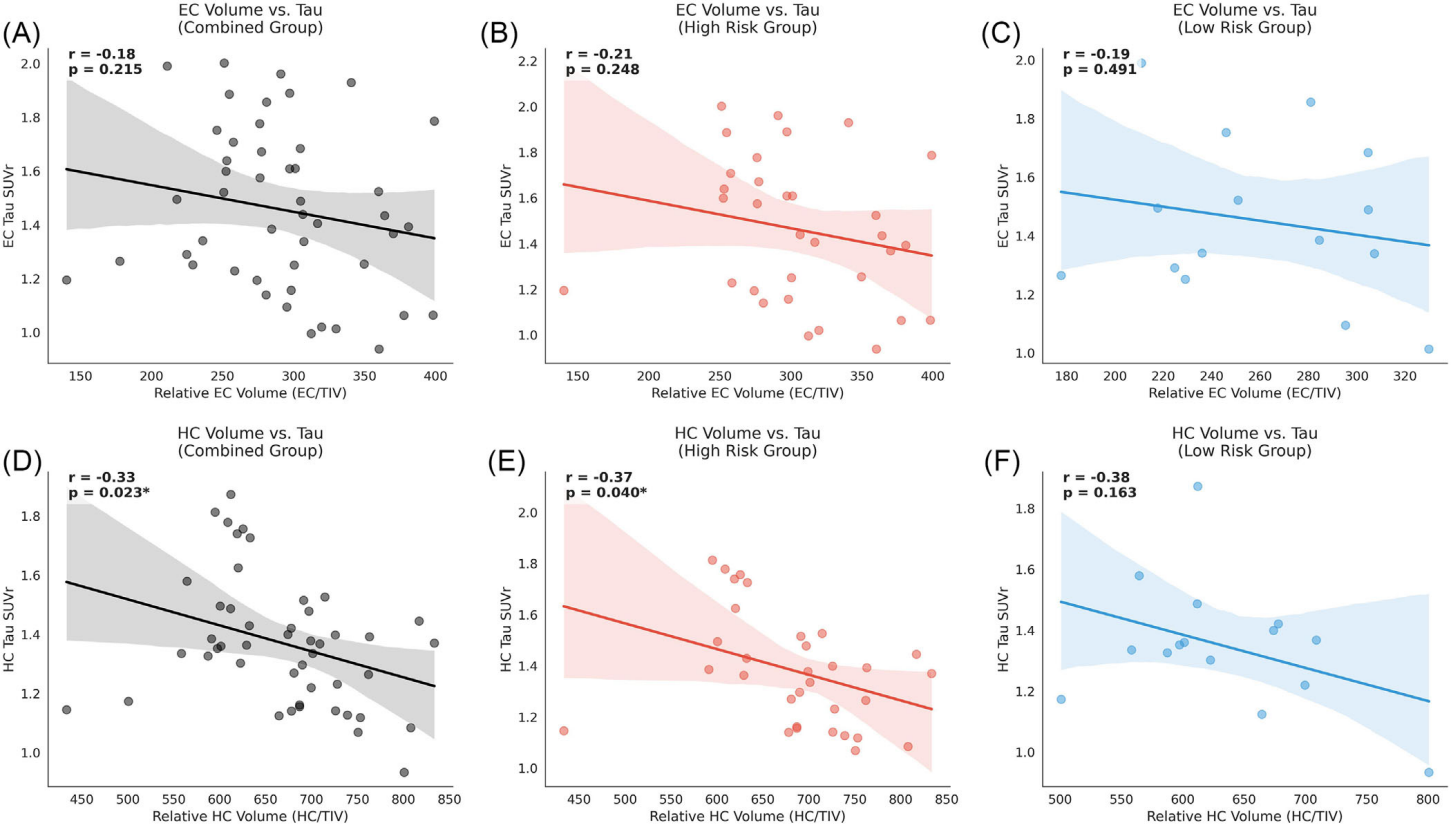
Correlation between relative entorhinal volume and tau SUVR in EC (A, B, C), correlation between relative hippocampal volume and tau SUVR in HC (D, E, F). EC, entorhinal cortex; HC, hippocampus; SUVR, standardized uptake value ratio; TIV, total intracranial volume.

### 3.4 Multivariate logistic regression: predicting MCI‐to‐AD conversion with anatomical stratification and tauopathy

We modeled the within 36 months MCI‐to‐AD conversion using multivariate logistic regression (Tables 4 and 5) in the overall cohort, incorporating demographic data, MMSE scores, relative EC and HC volumes, regional tau PET SUVR, and lateral space proximity indices. The model was statistically significant (LLR *P* = 0.012) with moderate explanatory power (pseudo *R* ^2^ = 0.323). The log‐likelihood of the fitted model compared to the null model improved from –32.567 to –22.043.

**TABLE 4:** Logistic model in combined group: conversion ∼ age + sex + MMSE + HC/TIV ratio + tau SUVR in HC + EC/TIV ratio + tau SUVR in EC + left space proximity index + right space proximity index.

	Regression model
Pseudo *R* ^2^	0.323
Log likelihood	−22.043
LL‐null	−32.567
LLR *P* value	<0.05 (0.012)

Abbreviations: EC, entorhinal cortex; HC, hippocampus; LL‐null, log‐likelihood of the null model; LLR, log‐likelihood ratio; MMSE, Mini‐Mental State Examination; SUVR, standardized uptake value ratio; TIV, total intracranial volume.

**TABLE 5:** Estimated coefficients for the predictors to predict conversion from MCI to AD.

Variables	Coeff.(β)	SE	*Z* value	*p* value
Constant	−4.33	7.73	−0.56	0.576
Age	−0.02	0.05	−0.33	0.739
Sex	1.42	1.02	1.39	0.165
MMSE	−0.02	0.18	−0.13	0.894
HC/TIV ratio	−0.01	0.006	−1.51	0.131
Tau SUVR in HC	−5.47	3.49	−1.57	0.117
EC/TIV Ratio	0.03	0.01	2.60	<0.01 (0.0094)
Tau SUVR in EC	7.91	2.97	2.66	<0.01 (0.0078)
Left space proximity index	−0.02	0.01	−2.20	<0.05 (0.0279)
Right space proximity index	0.02	0.008	2.28	<0.05 (0.0224)

Abbreviations: AD, Alzheimer's disease; Coeff.(β), regression coefficient; EC, entorhinal cortex; HC, hippocampus; MCI, mild cognitive impairment; MMSE, Mini‐Mental State Examination; PET, positron emission tomography; SE, standard error; SUVR, standardized uptake value ratio; TIV, total intracranial volume.

Age, sex, MMSE scores, relative hippocampal ratio, and HC tau SUVR were not significant predictors (*P* > 0.1 for all). In contrast, entorhinal tau SUVR significantly predicted the MCI‐to‐AD conversion, emphasizing the link between tauopathy and AD progression. The EC/TIV ratio was also significant (β = 0.03, *P* = 0.0094), indicating that greater relative EC volume was associated with increased MCI‐to‐AD conversion risk. It is worth noting that the left and right space proximity indices showed opposite effects in the regression. Left NCC was associated with reduced conversion risk (β = –0.02, *P* = 0.028), while right proximity was associated with increased risk (β = 0.02, *P* = 0.022).

To assess whether the EC anatomical risk classification modulates other predictors of conversion, we performed logistic regression in high and low EC anatomical risk groups. The first model excluded hippocampal metrics (Tables 6 and 7), while the second included them (Tables 8 and 9).

**TABLE 6:** Logistic regression model in high risk versus low risk group: conversion ∼ age + sex + MMSE + EC/TIV ratio + tau SUVR in EC.

	Regression model in high channel space risk group	Regression model in low channel space risk group
Pseudo *R* ^2^	0.464	0.139
Log likelihood	−8.877	−13.704
LL‐null	−16.552	−15.921
LLR *P* value	<0.01 (0.009)	0.489

Abbreviations: EC, entorhinal cortex; LL‐null, log‐likelihood of the null model; LLR, log‐likelihood ratio; MMSE, Mini‐Mental State Examination; SUVR, standardized uptake value ratio; TIV, total intracranial volume.

**TABLE 7:** Estimated coefficients for the predictors to predict conversion from MCI to AD.

	Regression model in high channel space risk group				Regression model in low channel space risk group			
Variables	Coeff. (β)	SE	*Z* value	*p* value	Coeff. (β)	SE	*Z* value	*p* value
Constant	0.28	0.85	0.33	0.740	0.26	0.93	0.28	0.778
Age	0.15	0.69	0.22	0.828	−0.83	0.52	−1.60	0.109
Sex	0.49	1.29	0.38	0.704	−0.52	1.18	−0.45	0.656
MMSE	−1.25	1.02	−1.22	0.223	0.38	0.48	0.80	0.423
EC/TIV ratio	1.85	1.37	1.35	0.177	0.47	0.52	0.91	0.364
Tau PET SUVR in EC	2.66	1.27	2.10	<0.05 (0.036)	0.29	0.53	0.55	0.586

Abbreviations: AD, Alzheimer's disease; Coeff.(β), regression coefficient; EC, entorhinal cortex; MCI, mild cognitive impairment; MMSE, Mini‐Mental State Examination; PET, positron emission tomography; SE, standard error; SUVR, standardized uptake value ratio; TIV, total intracranial volume.

**TABLE 8:** Logistic regression model in high risk versus low risk group: conversion ∼ age + sex + MMSE + HC/TIV ratio + tau SUVR in HC + EC/TIV ratio + tau SUVR in EC.

	Regression model in high channel space risk group	Regression model in low channel space risk group
Pseudo *R* ^2^	0.579	0.476
Log likelihood	−6.963	−8.351
LL‐null	−16.552	−15.921
LLR *P* value	<0.01 (0.008)	<0.05 (0.03)

Abbreviations: EC, entorhinal cortex; HC, hippocampus; LL‐null, log‐likelihood of the null model; LLR, log‐likelihood ratio; MMSE, Mini‐Mental State Examination; SUVR, standardized uptake value ratio; TIV, total intracranial volume.

**TABLE 9:** Estimated coefficients for the predictors to predict conversion from MCI to AD.

	Regression model in high channel space risk group				Regression model in low channel space risk group			
Variables	Coeff. (β)	SE	*Z* value	*p* value	Coeff. (β)	SE	*Z* value	*p* value
Constant	0.43	0.95	0.45	0.655	−0.64	1.17	−0.55	0.582
Age	1.01	0.85	1.19	0.234	−2.04	1.11	−1.85	0.065
Sex	0.35	1.59	0.22	0.826	1.00	1.74	0.58	0.565
MMSE	−0.50	0.94	−0.53	0.594	0.85	0.61	1.40	0.162
HC/TIV ratio	3.03	2.50	1.21	0.226	−3.35	1.61	−2.08	<0.05 (0.037)
Tau PET SUVR in HC	−2.01	1.82	−1.11	0.268	−1.37	1.06	−1.30	0.195
EC/TIV ratio	0.28	1.52	0.19	0.852	2.96	1.63	1.82	0.068
Tau PET SUVR in EC	5.80	3.02	1.92	0.054	1.38	0.98	1.42	0.156

Abbreviations: AD, Alzheimer's disease; Coeff.(β), regression coefficient; EC, entorhinal cortex; HC, hippocampus; MCI, mild cognitive impairment; MMSE, Mini‐Mental State Examination; PET, positron emission tomography; SE, standard error; SUVR, standardized uptake value ratio; TIV, total intracranial volume.

Strikingly, EC tau burden could only positively predict MCI‐to‐AD conversion only among participants classified as high anatomical risk. In this group, the first model was significant (pseudo *R* ^2^ = 0.464; LLR *P* = 0.009), with EC tau SUVR emerging as the only significant predictor (β = 2.66, *P* = 0.036). Other variables, including age, sex, MMSE scores, and EC/TIV ratio, were not significant predictors. In contrast, the model was not significant in the low‐risk group (pseudo *R* ^2^ = 0.139; LLR *P* = 0.489), and no predictors reached significance. Additionally, to address risk of Type I errors due to our predictors, we performed this analysis with reduced predictors (Table S3–S5 in supporting information) as well as performing an internal validation using 1000 bootstrap resamples on the parameter reduced model (Table S6 in supporting information), as well as including a Firth bias reduction, finding the effect of conversion in the high‐risk group to remain stable and significant.

Adding hippocampal metrics improved prediction in both the high‐ (pseudo *R* ^2^ = 0.579; LLR *P* = 0.008) and low‐risk group (pseudo *R* ^2^ = 0.476; LLR *P* = 0.03). In the high‐risk group, EC tau SUVR showed a trend toward significance (β = 5.80, *P* = 0.054), while hippocampal variables remained non‐significant while, notably, in the low‐risk group, the HC/TIV ratio was significant (β = −3.35, *P* = 0.037), indicating smaller hippocampal volumes associated with higher risk of conversion.

We also modeled conversion using tau SUVR in the EC as the sole predictor; the model was not significant for either the overall cohort (Figure S8 in supporting information) or the low‐risk group (Figure S9 in supporting information). Consistent with the multivariable logistic regression results described above, tau SUVR in the EC significantly predicted MCI‐to‐AD conversion only within the high‐risk group (Figure S9). To account for other established AD etiologies, we performed supplementary analyses adjusting for Aβ status (Table S7 in supporting information). In addition, to evaluate the impact of different morphometric measures, we conducted supplemental comparison of lateral EC cortical thickness between high‐ and low‐risk groups (Table S8 in supporting information), and modeling using EC cortical thickness as a covariate in place of relative EC volume (Table S9 in supporting information). Last, to address effects of genetic causal links and our finding, we included apolipoprotein E (*APOE*) ε4 carrier status and load in a reduced model finding that tau EC and its relationship to conversion remain unchanged (Tables S10–S11 in supporting information).

### 3.5 Sensitivity analysis

To ensure the robustness of our findings, we performed a sensitivity analysis by comparing demographic, imaging statistics, and logistic regression models mentioned above with and without the identified outlier (Figures S10–S19 and Tables S12–S18 in supporting information). The predictive value of EC tau SUVR in the high‐risk group remained significant in both iterations, while the models remained not significant in the overall cohort and the low‐risk group, confirming that the association was not driven by extreme values.

### 3.6 Heuristic cutoff derivation validation

To address the heuristic nature of our cutoff of 500, in a reduced model (with Firth reduction bias), we reperformed our analysis using groups of both NCC = 400, 450, 500, and 550, all preserving our conclusion. In our original model (with Firth reduction bias), NCC = 450, 500, 550, and 600 preserve our conclusion (Tables S19–S20 in supporting information). Both ranges contain and center around 500, our chosen value. Further studies should address the unique morphological shape of this region given that it is radiologically defined in vivo and the full complex of characteristics have yet to be fully explored.

## 4 DISCUSSION

In this study, we are the first to directly evaluate the contribution of chronic mechanical stress (through abnormal tau burden in the EC) and its role in AD initiation and progression using multimodal neuroimaging and topographical radiological measures. We find that EC tau burden predicted conversion to AD specifically among individuals with high EC–TI proximity, but not among those with low proximity. We hypothesize that continual EC–TI contact may trigger mechanical stress. This may contribute to neurofibrillary degeneration, ultimately increasing the likelihood of acceleration or progression of individuals at high risk of developing AD in concert with the intrinsic accumulation of NFTs. We examine this through an analysis of individuals with MCI with and without prodromal AD dementia, with both structural MRI and tau PET imaging. We were able to quantify this channel, which we refer to as the “uncal fossa,” defining risk by adjacency. We further examine other AD‐related clinical, imaging, and biofluidic measures. Within this group, our measure of adjacency heuristically split the initial MCI group relatively evenly. There is considerable variance in adult brain and skull physiology and anatomy^28^ and, to our knowledge, this area has yet to be studied or defined.

TBI has been associated with elevated risk of developing AD,^29^ with tau pathology distribution following patterns similar to those found in AD.^30^, ^31^ In a study examining individuals with MCI/AD impairment with and without a history of head impact (HI), elevated tau PET was seen in frontal and parietal lobes in those with a HI.^32^ Unlike CTE, which originates from neocortical perivascular spaces (not necessarily targeting medial temporal structures), HI reflects a single event.^33^ We suspect that tauopathic changes due to HI in MCI/AD are separate from those from the chronic subclinical microtrauma (including potential microcerebrovascular impairments) that comes from the surface of the EC near the TI in high‐risk individuals. Computational models of head impacts imply heterogeneity of the cortical surface morphology, including considerations of the boundary conditions imposed by adjacent rigid structures (like the TI) which may significantly modulate the local distribution of mechanical stress and strain,^34^, ^35^ and evidence of the skull–brain interface contributing to injury, including through its neurovascular and neuroimmune consequences.^36^ However, such risks could be cumulative, in addition to the types of mechanisms seen in TBI. Analogously, hyperphosphorylated tau accumulation in National Football League players has been linked to monotonic overloads and cyclic fatigue or “creep”;^37^ by contrast, high EC/TI proximity implies far lower impulse at far higher frequency, where even isolated “jolts” may suffice.

Mechanically, one way to reduce exposure of a planar surface to an abutting coarse structure is to include or place prominences on that surface;^38^ the EC is the one area with such prominences, called “verrucae.” These verrucae are thought to originate in layer II of the EC^39^, ^40^ and mature across adulthood. This postnatal developmental timeline, unlike gyrification for instance, raises the possibility that verrucae represent an evolutionary adaptation to potential chronic low‐level mechanical forces, analogous to callus tissue formation of connective tissue in areas undergoing repetitive loading. Augustinack et al. previously reported that verrucae size declines with advancing Braak stage,^39^ specifically with layer II entorhinal neuron loss, a site of earliest tau vulnerability. We propose that these raised structures might serve an evolutionary and mechanistic function in reducing potential contact between a sensitive and important structure (the EC) and a stiff surface (the TI), or alternatively, these prominences being particularly susceptible to subclinical compression may accelerate a cascade of substantially focal tau accumulation. These interpretations yield testable predictions: if verrucae are protective, higher density should correspond to reduced tau burden relative to EC–TI proximity, whereas if they represent particular vulnerability, higher density should correlate with surface‐level tau, with high‐adjacency individuals at increased risk. Multimodal imaging, such as combining MR elastography and high‐resolution structural imaging to resolve verrucae morphology, tau burden, and EC–TI proximity, may help address this.

There is a predicate for singular structures of the brain being more vulnerable to injury on the basis of their isolated location. For instance, the anterior cingulate is bifurcated bilaterally by the stiff falx cerebri and subfalcine herniations result from the cingulate gyrus extending around its stiff sickle‐shaped dura.^1^ Reduced performance and reduced brain volume have been reported in individuals with known TBI compared to controls.^41^ Therefore, vulnerability to a stiff partition can yield functional and structural consequences.

This study has important limitations in understanding the time of MRI with respect to disease course, degree of suspected impairment, and extrinsic contributing factors of tau burden. It is not clear where specifically along the medio‐lateral aspect of the EC tau accumulation occurs relative to its adjacency to the TI. According to Braak staging, the first evidence of NFT pathology in the EC is the lateral aspect of the EC, which is likely less proximal to the TI than the medial.^8^ There may be several factors that are responsible for the initiation of EC‐related tau accumulation and eventual spread.^42^ Relatedly, we did not stratify our population according to spatial tau or metabolic patterns. We posit that any contribution of a high EC–TI adjacency would have a greater impact on limbic‐predominant disease compared to ones in which the hippocampus is spared.^43^, ^44^, ^45^ The presence of potential tau accumulation resulting from proximity to the TI within the EC may be additive and not exclusive to the initial deposition of tau in the lateral EC/transentorhinal cortex (TEC). Although *APOE* ε4 status did not impact the result or channel distance, we cannot exclude other known AD genetic mutations from impacting this result. As such, areas of tau spread, to both the hippocampus and external cortical areas, may be accelerated by the total tau burden within the entire EC circuit.^46^ Our findings support this because EC/TI adjacency alone did not significantly predict conversion of MCI to AD; however, evidence of tau accumulation within the high‐adjacency group did, indicating that other factors are dominant in establishing likelihood of progression in this population. Paradoxically, high adjacency suggests a thicker EC, in contrast to the relatively thinner EC that is seen in those who ultimately go on to develop AD.^47^ We found opposing hemispheric associations in our analysis, with left NCC associated with reduced conversion risk and right NCC with increased risk. It is possible that this reflects a well‐documented hemispheric asymmetry of tau pathology in AD, which has been tracked from early and prodromal disease stages and corresponds to hemispheric amyloid deposition.^48^ The tentorial notch may, itself, be anatomically asymmetric across individuals^21^ and the forces differentially imparted on each EC may vary by hemisphere. Exploration into laterality and sufficiency will require more detailed and hemispheric‐centered analyses. Additionally, hippocampal volume was not predictive of progression in our MCI group, indicating that obvious structural MRI findings, likely reflective of sufficient neuronal loss, are not sufficient to augur conversion to AD.

Building upon our findings, we propose a “biomechanical cascade hypothesis” in which chronic low‐level mechanical stress acts as an initiating or accelerating factor in tau pathology. Analogous to orthopedic and cardiovascular pathology, “progressive mechanical failure” of tissue can cause redistributed loading and downstream damage, a plausible and under‐recognized mechanism in neurodegeneration. This reflects mechanisms in other systems, such as intervertebral disc degeneration and subsequent maladaptive stress redistribution that hastens degeneration,^49^ the tears in rotator cuff leading to cascading failures and shoulder instability^50^ and anterior cruciate ligament tears and knee destabilization leading to cartilage damage.^51^ Even in cardiovascular failure, damage to a localized region of myocardium can result in maladaptive remodeling and decline.^52^ These human physiological examples share the theme of initial localized failure leading to progressive dysfunction through biomechanical propagation.

Applying this paradigm to the brain, the EC may either trigger or exacerbate tau accumulation. This mechanical nidus could initiate a cascade of localized tau seeding, altered neural connectivity, metabolic stress, and eventual tauopathic spread, in which variability may further modulate the degree of stress imparted on adjacent structures.^21^ A narrower or asymmetric tentorial opening may amplify this, particularly in scenarios of minor trauma, brain shift, or aging‐related atrophy. Similar biomechanical principles could underlie other sporadic neurodegenerative diseases. For example, in Parkinson's disease, the substantia nigra lies near a junction vulnerable to rotational shear forces,^53^ while in progressive supranuclear palsy and corticobasal degeneration, both tauopathies, the pathology may reflect local strain of midline and subcortical structures.^54^ This suggests a confluence of constitutional anatomical configuration, vulnerability, and chronic micro‐injury.

While other mechanisms, such as inflammation,^55^ excitotoxicity,^56^ or viral agents,^57^ are known contributors to tauopathy, the paucity of definitive data establishing them in early entorhinal tauopathy strengthens the case for mechanical injury as a plausible alternative root cause or amplifier. Notably, tau accumulation does not commonly occur in purely inflammatory or viral brain diseases, raising the possibility that a non‐immunologic physical mechanism may contribute to the pathogenesis of certain tauopathies, though this inference extends beyond the evidence here and warrants further clinical and mechanistic investigation. Regardless, the convergence of anatomical vulnerability, biomechanical strain, and tau pathology warrants deeper exploration in imaging, *post mortem*, and experimental models.

## CONFLICT OF INTEREST STATEMENT

The authors report no relevant conflicts of interest pertaining to this manuscript. Author disclosures are available in the Supporting Information.

## CONSENT STATEMENT

All human subjects data used in this study were obtained from the Alzheimer's Disease Neuroimaging Initiative (ADNI) database (adni.loni.usc.edu). Written informed consent was obtained from all participants or their authorized representatives at each ADNI site at the time of enrollment. The ADNI study was approved by the institutional review board (IRB) at each participating institution, and all procedures were performed in accordance with the Declaration of Helsinki.

## Supporting information



Supporting Information

Supporting Information
