# Supplementary material for: Structural compression and entorhinal vulnerability: Linking tentorial adjacency to tau burden and dementia progression
Source: Alzheimers Dement. 2026 Oct 5;22(10):e71827. doi: 10.1002/alz.71827 (PMC13636655; doi:10.1002/alz.71827)
Supplement: Supplementary file 2 [file ALZ-22-e71827-s002.docx]

# SUPPLEMENTAL MATERIALS

## Matlab, Python scripts used and raw data files are archived at <https://github.com/LyZhang502/Chronic_EC_Compression.git>

## Supplemental Figures on the Full Data

Figure S1. Data Distribution in High Risk Group

Figure S2. Data Distribution in Low Risk Group

Figure S3. Correlation between Left Space Index (NCC) and Left EC tau PET SUVr

Figure S4. Correlation between Right Space Index (NCC) and Right EC tau PET SUVr

Figure S5. Correlation between relative entorhinal volume and space index (NCC): Significant correlation between left/right relative entorhinal volume and left/right space index in combined and low-risk group, but not in the high-risk group

Figure S6. Correlation between entorhinal cortical thickness and space index (NCC): Significant correlation between left/right entorhinal cortical thickness and left/right space index in combined and low-risk group, but not in the high-risk group

Figure S7. Correlation between entorhinal cortical thickness and entorhinal tau PET SUVr

Figure S8. Logistic Regression (Conversion ~ tau SUVr in EC) in the Combined group, the model is not significant, and tau SUVr in EC cannot significantly predict the conversion from MCI to AD

Figure S9. Logistic Regression (Conversion ~ tau SUVr in EC) in High Risk Group and Low Risk Group, the model is only significant in high risk group (LLR p < 0.01), and tau SUVr in EC can only predict conversion from MCI to AD positively in high risk group (p =0.0106)

## Supplemental Figures on the Data without the Outlier

There is one outlier in the space index in the dataset, the outlier subject is categorized as “Low Risk”. The following figures are the same analysis but on the dataset without the outlier.

Figure S10. Data Distribution of High Risk Group (without outlier)

Figure S11. Data Distribution of Low Risk Group (without outlier)

Figure S12. Correlation between Relative EC Volume and tau SUVr in EC in Combined Group, High Risk Group and Low Risk Group (without outlier)

Figure S13. Correlation between Relative HC Volume and tau SUVr in EC in Combined Group, High Risk Group and Low Risk Group (without outlier)

Figure S14. Correlation between Left Space Index and tau PET SUVr in Left EC (without outlier)

Figure S15. Correlation between Right Space Index and tau PET SUVr in Right EC (without outlier)

Figure S16. Correlation between Lateral Space Index and Lateral EC Cortical Thickness in Combined Group, High Risk Group and Low Risk Group (without outlier)

Figure S17. Correlation between Lateral EC Cortical Thickness and tau SUVr in lateral EC in Combined Group, High Risk Group and Low Risk Group (without outlier)

Figure S18. Logistic Regression (Conversion ~ tau SUVr in EC) in the Combined group without outlier, the model is not significant, and tau SUVr in EC cannot significantly predict the conversion from MCI to AD

Figure S19. Logistic Regression (Conversion ~ tau SUVr in EC) in High Risk Group and Low Risk Group without outlier, the model is only significant in high risk group (LLR p < 0.01), and tau SUVr in EC can only predict conversion from MCI to AD positively in high risk group (p =0.0106)

## Supplemental Tables

Table S1. Repeated Manual Segmentation Adjustment of EC-TI Channel Dice Reliability

| Subject | Run 1 voxels | Run 2 voxels | Intersection | Dice | Jaccard |
| --- | --- | --- | --- | --- | --- |
| 003_S_6268 | 155 | 147 | 145 | 0.960 | 0.924 |
| 023_S_6356 | 512 | 508 | 501 | 0.982 | 0.965 |
| 032_S_6700 | 186 | 189 | 182 | 0.971 | 0.943 |
| 070_S_6911 | 367 | 365 | 354 | 0.967 | 0.937 |
| 135_S_6703 | 200 | 197 | 190 | 0.957 | 0.918 |

Table S2. Inclusion of Different FreeSurfer Versions (FreeSurfer 7.3 v.s. FreeSurfer 6.0) as a Predictor in Reduced Regression Model with Firth’s Bias Reduction (Conversion ~ MMSE + tau SUVr in EC + ADNI4_MRI_epoch)

| Dataset | Group | β (EC tau) | p (EC tau) | β (ADNI4_MRI_epoch) | p (ADNI4_MRI_epoch) |
| --- | --- | --- | --- | --- | --- |
| Full Dataset  (Firth) | High Risk (n=24) | 1.60 | 0.028 | -0.95 | 0.173 |
|  | Low Risk (n=23) | 0.26 | 0.564 | 0.09 | 0.838 |
| Without Outlier (Firth) | High Risk (n=24) | 1.60 | 0.028 | -0.95 | 0.173 |
|  | Low Risk (n=22) | 0.23 | 0.610 | 0.24 | 0.590 |

ADNI4 uses FreeSurfer 7.3, while ADNI3 uses FreeSurfer 6.0. We retrieved the scan time of baseline T1w MR scans and defined the dummy variable ADNI4_MRI_epoch=1 if the scan date is equal to or later than Jan 1^st^, 2021, otherwise the variable is defined as 0.

Table S3. Reduced Covariate Logistic Regression Models (Conversion ~ tau SUVr in EC + MMSE) with Regular (MLE) Estimator

| Dataset | Group | β(tau SUVr in EC) | p-value (β- tau SUVr in EC) |
| --- | --- | --- | --- |
| Full Dataset | Combined (n=47) | 0.83 | 0.018 |
|  | High Risk (n=24) | 1.68 | 0.013 |
|  | Low Risk (n=23) | 0.28 | 0.518 |
| Without Outlier | Combined (n=46) | 0.80 | 0.022 |
|  | High Risk (n=24) | 1.68 | 0.013 |
|  | Low Risk (n=22) | 0.22 | 0.619 |

Table S4. Reduced Covariate Logistic Regression Models (Conversion ~ tau SUVr in EC + MMSE) with Firth’s Bias Reduction (for Small Sample Size)

| Dataset | Group | β(tau SUVr in EC) | p-value (β- tau SUVr in EC) |
| --- | --- | --- | --- |
| Full Dataset | Combined (n=47) | 0.76 | 0.026 |
|  | High Risk (n=24) | 1.33 | 0.022 |
|  | Low Risk (n=23) | 0.24 | 0.576 |
| Without Outlier | Combined (n=46) | 0.73 | 0.032 |
|  | High Risk (n=24) | 1.33 | 0.022 |
|  | Low Risk (n=22) | 0.19 | 0.671 |

Table S5. Original Logistic Regression Models (Conversion ~ tau SUVr in EC + MMSE + Age + Sex + EC/TIV Ratio) with Firth’s Bias Reduction

| Dataset (Estimator) | Group | β(EC tau SUVr) | p-value (β-EC tau SUVr) |
| --- | --- | --- | --- |
| Full Dataset (Firth) | Combined (n=47) | 0.86 | 0.019 |
|  | High Risk (n=24) | 1.42 | 0.035 |
|  | Low Risk (n=23) | 0.22 | 0.673 |
| Without Outlier (Firth) | Combined (n=46) | 0.85 | 0.023 |
|  | High Risk (n=24) | 1.42 | 0.035 |
|  | Low Risk (n=22) | 0.17 | 0.742 |

Table S6. Bootstrapping Validation for Reduced Logistic Regression Models (Conversion ~ tau SUVr in EC + MMSE) with and without Firth’s Bias Reduction (1000 successful resampling permutations, random seed: 20260611)

| Dataset | Estimator | Group | Bootstrapped β (tau SUVr in EC) 95% CI | Apparent AUC | Optimism-corrected AUC |
| --- | --- | --- | --- | --- | --- |
| Full Dataset | Regular (MLE) | Combined (n=47) | 0.27, 1.94 | 0.726 | 0.694 |
|  |  | High Risk (n=24) | 0.74, 255.47 | 0.874 | 0.837 |
|  |  | Low Risk (n=23) | -0.64, 2.49 | 0.583 | 0.455 |
|  | Firth | Combined (n=47) | 0.20, 1.84 | 0.728 | 0.697 |
|  |  | High Risk (n=24) | 0.57, 4.57 | 0.881 | 0.847 |
|  |  | Low Risk (n=23) | -0.55, 1.43 | 0.583 | 0.456 |
| Without Outlier | Regular (MLE) | Combined (n=46) | 0.11, 2.10 | 0.718 | 0.684 |
|  |  | High Risk (n=24) | 0.70, 284.51 | 0.874 | 0.836 |
|  |  | Low Risk (n=22) | -0.73, 2.03 | 0.579 | 0.437 |
|  | Firth | Combined (n=46) | 0.14, 1.74 | 0.718 | 0.682 |
|  |  | High Risk (n=24) | 0.58, 4.87 | 0.881 | 0.849 |
|  |  | Low Risk (n=22) | -0.59, 1.46 | 0.562 | 0.424 |

Table S7. Logistic Regression Model (Conversion ~ Age + Sex + MMSE + Amyloid Status + EC/TIV Ratio + tau PET SUVr in EC) in High and Low Risk Group, taking amyloid status into consideration

|  | Regression Model in High Channel Space Risk Group |
| --- | --- |
| Pseudo R-square | 0.4934 |
| Log Likelihood | -8.3858 |
| LL-Null | -16.552 |
| LLR p-value | 0.01208* |

| Variables | Regression Model in High Channel Space Risk Group | | | | Regression Model in Low Channel Space Risk Group | | | |
| --- | --- | --- | --- | --- | --- | --- | --- | --- |
|  | Coeff. (β) | Std. Err. | z | p | Coeff. (β) | Std. Err. | z | p |
| Const. | 2.57 | 2.77 | 0.93 | 0.354 | -0.35 | 1.52 | -0.23 | 0.817 |
| Age | 0.21 | 0.78 | 0.27 | 0.790 | -0.84 | 0.52 | -1.60 | 0.109 |
| Sex | 0.28 | 1.37 | 0.20 | 0.839 | -0.40 | 1.20 | -0.34 | 0.736 |
| MMSE | -0.80 | 1.10 | -0.72 | 0.470 | 0.39 | 0.47 | 0.83 | 0.406 |
| Amyloid status (binary) | -3.08 | 3.41 | -0.90 | 0.367 | 0.69 | 1.34 | 0.51 | 0.610 |
| EC/TIV Ratio | 2.61 | 1.68 | 1.55 | 0.120 | 0.48 | 0.53 | 0.89 | 0.372 |
| tau PET SUVr in EC | 4.07 | 2.19 | 1.86 | 0.063 | 0.17 | 0.58 | 0.29 | 0.770 |

Table S8. Cortical Thickness Comparison between High-Risk and Low-Risk Groups

|  | High Channel Space Risk (n=24) | Low Channel Space Risk (n=23) | t test statistics | High Risk *vs*. Low Risk *Sig.* |
| --- | --- | --- | --- | --- |
| Left Entorhinal Cortical Thickness | 2.991 ± 0.410 | 2.788 ± 0.465 | t = 1.5921 | p = 0.12 |
| Right Entorhinal Cortical Thickness | 2.971 ± 0.378 | 2.920 ± 0.485 | t = 0.4051 | p = 0.69 |

Table S9. Logistic Regression Model (Conversion ~ Age + Sex + MMSE + HC/TIV Ratio + tau SUVr in HC + Left EC Cortical Thickness + Right EC Cortical Thickness + tau SUVr in EC + Left Space Proximity Index + Right Space Proximity Index), EC Cortical Thickness Replacing Relative EC Volume as a Covariate

|  | Regression Model |
| --- | --- |
| Pseudo R-square | 0.2075 |
| Log Likelihood | -25.810 |
| LL-Null | -32.567 |
| LLR p-value | 0.1963 |

| Variables | Coeff.(β) | Std. Err. | z | p |
| --- | --- | --- | --- | --- |
| Const. | -8.0969 | 7.992 | -1.013 | 0.311 |
| Age | 0.0319 | 0.052 | 0.615 | 0.538 |
| Sex | 0.6359 | 0.883 | 0.720 | 0.472 |
| MMSE | 0.0153 | 0.166 | 0.093 | 0.926 |
| HC/TIV Ratio | 0.0029 | 0.005 | 0.548 | 0.584 |
| tau SUVr in HC | -3.5318 | 3.008 | -1.174 | 0.240 |
| Left EC Cortical Thickness | -0.8805 | 1.183 | -0.744 | 0.457 |
| Right EC Cortical Thickness | 1.0155 | 1.262 | 0.805 | 0.421 |
| tau SUVr in EC | 5.6277 | 2.516 | 2.237 | 0.025^*^ |
| Left Space Proximity Index | -0.0152 | 0.008 | -1.827 | 0.068 |
| Right Space Proximity Index | 0.0112 | 0.008 | 1.481 | 0.139 |

Table S10. ApoE4 Allele Load-adjusted Logistic Regression Models (Conversion ~ ApoE4 Allele Load + MMSE + tau SUVr in EC) with Firth’s Bias Reduction

| Dataset | Group | β (EC tau) | p (EC tau) | β (ApoE4) | p (ApoE4) |
| --- | --- | --- | --- | --- | --- |
| Full Dataset  (Firth) | Combined (n=46/47) | 0.98 | 0.014 | -0.57 | 0.136 |
|  | High Risk (n=24/24) | 1.84 | 0.026 | -0.66 | 0.306 |
|  | Low Risk (n=22/23) | 0.28 | 0.544 | -0.74 | 0.166 |
| Without Outlier (Firth) | Combined (n=45/46) | 0.96 | 0.016 | -0.61 | 0.118 |
|  | High Risk (n=24/24) | 1.84 | 0.026 | -0.66 | 0.306 |
|  | Low Risk (n=21/22) | 0.21 | 0.658 | -0.85 | 0.127 |

There is one missing ApoE data (SubjectID: 033_S_7088), here we applied the complete-case analysis.

Table S11. ApoE4 Carrier Status adjusted Logistic Regression Models (Conversion ~ ApoE4 Carrier Status + MMSE + tau SUVr in EC) with Firth’s Bias Reduction

| Dataset | Group | β (EC tau) | p (EC tau) | β (ApoE4) | p (ApoE4) |
| --- | --- | --- | --- | --- | --- |
| Full Dataset  (Firth) | Combined (n=46/47) | 0.83 | 0.028 | -0.23 | 0.517 |
|  | High Risk (n=24/24) | 1.49 | 0.049 | -0.27 | 0.676 |
|  | Low Risk (n=22/23) | 0.25 | 0.587 | -0.52 | 0.262 |
| Without Outlier (Firth) | Combined (n=45/46) | 0.82 | 0.031 | -0.26 | 0.459 |
|  | High Risk (n=24/24) | 1.49 | 0.049 | -0.27 | 0.676 |
|  | Low Risk (n=21/22) | 0.18 | 0.709 | -0.62 | 0.195 |

There is one missing ApoE data (SubjectID: 033_S_7088), here we applied the complete-case analysis.

Table S12. Demographic Data, Clinical Assessment, and Amyloid Status of Participants that Did or Did Not Convert from MCI to AD without the Outlier

|  | Converted to AD within 36 Months (n=24) | Did Not Convert to AD within 36 Months (n=22) | t test or Chi-square test statistics | Converted vs. Not Converted Sig. |
| --- | --- | --- | --- | --- |
| Age (Baseline,yrs) | 74.13 ± 7.71 | 74.19 ± 7.77 | t = -0.0252 | p = 0.9800 |
| MMSE | 26.46 ± 2.34 | 26.77 ± 2.27 | t = -0.4621 | p = 0.6463 |
| Sex (women, %) | 50 | 59.09 | Chi = 0.1037 | p = 0.7474 |
| Amyloid Status (Positive, %) | 83.33 | 59.09 | Chi = 2.2390 | p = 0.1346 |

Table S13. Demographic Data, Clinical Assessment, Amyloid Status, Left and Right Space Proximity Index and Conversion Rates of Participants (without the Outlier)

|  | High Channel Space Risk (n=24) | Low Channel Space Risk (n=22) | t test or Chi-square test statistics | Converted vs. Not Converted Sig. |
| --- | --- | --- | --- | --- |
| Age | 73.45 ± 8.04 | 74.94 ± 7.32 | t = -0.6577 | p = 0.5142 |
| Sex (women, %) | 58.33 | 68.18 | Chi = 2.2716 | p = 0.1318 |
| MMSE | 26.83 ± 1.83 | 26.36 ± 2.72 | t = 0.6924 | p = 0.4923 |
| Amyloid Status (Positive, %) | 66.67 | 77.27 | Chi = 0.2212 | p = 0.6382 |
| Left Space Proximity Index | 44.90 ± 30.04 | 149.62 ± 63.67 | t = -7.2320 | p = 0.0000* |
| Right Space Proximity Index | 32.98 ± 24.42 | 105.91 ± 74.93 | t = -4.5177 | p = 0.0000* |
| Conversion (Positive, %) | 54.17 | 50 | Chi = 0.0000 | p = 1.0000 |

Table S14. Imaging Metrics Comparison between High Channel Risk Group and Low Channel Risk Group without the Outlier (EC: entorhinal cortex; LEC: left entorhinal cortex; REC: right entorhinal cortex; HC: hippocampus; LHC: left hippocampus; RHC: right hippocampus; TIV: total intracranial volume; PET: Positron emission tomography; SUVr: Standardized Uptake Value Ratio)

|  | High Channel Space Risk (n=24) | Low Channel Space Risk (n=22) | t test or Chi-square test statistics | Converted vs. Not Converted Sig. |
| --- | --- | --- | --- | --- |
| EC/TIV Ratio | 294.36 ± 50.54 | 293.22 ± 57.74 | t = 0.0709 | p = 0.9438 |
| LEC/TIV Ratio | 306.68 ± 62.89 | 296.36 ± 56.27 | t = 0.5849 | p = 0.5616 |
| REC/TIV Ratio | 282.03 ± 50.81 | 290.09 ± 78.40 | t = -0.4174 | p = 0.6784 |
| HC/TIV Ratio | 687.18 ± 88.60 | 645.71 ± 69.84 | t = 1.7520 | p = 0.0867 |
| LHC/TIV Ratio | 694.05 ± 98.40 | 622.09 ± 90.30 | t = 2.5769 | p = 0.0134* |
| RHC/TIV Ratio | 682.35 ± 111.38 | 675.55 ± 104.44 | t = 0.2132 | p = 0.8321 |
| tau PET SUVr in EC | 1.44 ± 0.30 | 1.48 ± 0.31 | t = -0.4884 | p = 0.6277 |
| tau PET SUVr in HC | 1.35 ± 0.22 | 1.40 ± 0.22 | t = -0.8153 | p = 0.4193 |

Table S15. Logistic Regression to Predict Conversion Possibility in Combined Group (Model: Conversion ~ Age + Sex + MMSE + HC/TIV Ratio + tau SUVr in HC + EC/TIV Ratio + tau SUVr in EC + Left Space Proximity Index + Right Space Proximity Index), note that the coefficients are the same with or without the outlier

|  | Regression Model |
| --- | --- |
| Pseudo R-square | 0.3077 |
| Log Likelihood | -22.043 |
| LL-Null | -31.841 |
| LLR p-value | 0.02057* |

| Variables | Coeff.(β) | Std. Err. | z | p |
| --- | --- | --- | --- | --- |
| Const. | -4.3311 | 7.7349 | -0.5599 | 0.5755 |
| Age | -0.0175 | 0.0524 | -0.3335 | 0.7387 |
| Sex | 1.4242 | 1.0253 | 1.389 | 0.1648 |
| MMSE | -0.0242 | 0.1808 | -0.1338 | 0.8936 |
| HC/TIV Ratio | -0.0099 | 0.0065 | -1.5097 | 0.1311 |
| tau SUVr in HC | -5.4701 | 3.4869 | -1.5688 | 0.1167 |
| EC/TIV Ratio | 0.0307 | 0.0118 | 2.599 | **0.0094*** |
| tau SUVr in EC | 7.912 | 2.9731 | 2.6612 | **0.0078*** |
| Left Space Proximity Index | -0.0234 | 0.0106 | -2.1983 | **0.0279*** |
| Right Space Proximity Index | 0.0192 | 0.0084 | 2.2842 | **0.0224*** |

Table S16. Logistic Regression to Predict Conversion Possibility in High Risk vs. Low Risk Group (Model: Conversion ~ Age + Sex + MMSE + EC/TIV Ratio + tau SUVr in EC) without the outlier

|  | Regression Model in High Channel Space Risk Group | Regression Model in Low Channel Space Risk Group |
| --- | --- | --- |
| Pseudo R-square | 0.4637 | 0.1151 |
| Log Likelihood | -8.8770 | -13.494 |
| LL-Null | -16.552 | -15.249 |
| LLR p-value | 0.008966* | 0.6217 |

| Variables | Regression Model in High Channel Space Risk Group | | | | Regression Model in Low Channel Space Risk Group | | | |
| --- | --- | --- | --- | --- | --- | --- | --- | --- |
|  | Coeff. (β) | Std. Err. | z | p | Coeff. (β) | Std. Err. | z | p |
| Const. | 0.28 | 0.85 | 0.33 | 0.740 | 0.37 | 0.92 | 0.40 | 0.690 |
| Age | 0.15 | 0.69 | 0.22 | 0.828 | -0.81 | 0.52 | -1.56 | 0.120 |
| Sex | 0.49 | 1.29 | 0.38 | 0.704 | -0.54 | 1.17 | -0.46 | 0.646 |
| MMSE | -1.25 | 1.02 | -1.22 | 0.223 | 0.37 | 0.48 | 0.76 | 0.445 |
| EC/TIV Ratio | 1.85 | 1.37 | 1.35 | 0.177 | 0.35 | 0.52 | 0.66 | 0.508 |
| tau PET SUVr in EC | 2.66 | 1.27 | 2.10 | **0.0362*** | 0.23 | 0.54 | 0.42 | 0.671 |

Table S17. Logistic Regression to Predict Conversion Possibility in High Risk vs. Low Risk Group (Model: Conversion ~ Age + Sex + MMSE + HC/TIV Ratio + tau SUVr in HC + EC/TIV Ratio + tau SUVr in EC) without the outlier

|  | Regression Model in High Channel Space Risk Group | Regression Model in Low Channel Space Risk Group |
| --- | --- | --- |
| Pseudo R-square | 0.5793 | 0.4524 |
| Log Likelihood | -6.9629 | -8.3505 |
| LL-Null | -16.552 | -15.249 |
| LLR p-value | 0.007646* | 0.05490 |

| Variables | Regression Model in High Channel Space Risk Group | | | | Regression Model in Low Channel Space Risk Group | | | |
| --- | --- | --- | --- | --- | --- | --- | --- | --- |
|  | Coeff. (β) | Std. Err. | z | p | Coeff. (β) | Std. Err. | z | p |
| Const. | 0.43 | 0.95 | 0.45 | 0.655 | -0.26 | 1.13 | -0.23 | 0.817 |
| Age | 1.01 | 0.85 | 1.19 | 0.234 | -2.08 | 1.13 | -1.84 | 0.065 |
| Sex | 0.35 | 1.59 | 0.22 | 0.826 | 1.00 | 1.74 | 0.58 | 0.565 |
| MMSE | -0.50 | 0.94 | -0.53 | 0.594 | 0.87 | 0.62 | 1.40 | 0.162 |
| HC/TIV Ratio | 3.03 | 2.50 | 1.21 | 0.226 | -3.37 | 1.62 | -2.08 | 0.038 |
| tau PET SUVr in HC | -2.01 | 1.82 | -1.11 | 0.268 | -1.38 | 1.06 | -1.30 | 0.195 |
| EC/TIV Ratio | 0.28 | 1.52 | 0.19 | 0.852 | 2.79 | 1.53 | 1.82 | 0.069 |
| tau PET SUVr in EC | 5.80 | 3.02 | 1.92 | 0.054 | 1.40 | 0.99 | 1.42 | 0.157 |

Table S18. Logistic Regression (Conversion ~ tau SUVr in EC) in Combined Group, High Risk Group and Low Risk Group without the Outlier

| Variables | Logistic Regression Results | | | |
| --- | --- | --- | --- | --- |
|  | Coeff. (β) | Std. Err. | z | p |
| Combined Group LLR p-value | 0.01258* | | | |
| High Risk Group LLR p-value | 0.001250* | | | |
| Low Risk Group LLR p-value | 0.6521 | | | |
| Combined Group tau PET SUVr in EC | 2.6651 | 1.147 | 2.323 | 0.020* |
| High Risk Group tau PET SUVr in EC | 1.6843 | 0.659 | 2.557 | 0.011* |
| Low Risk Group tau PET SUVr in EC | 0.1931 | 0.430 | 0.449 | 0.654 |

Table S19. NCC Discrete Cutoff Subgroup Sensitivity in Reduced Logistic Regression Models (Conversion ~ tau SUVr in EC + MMSE) with Firth’s Bias Reduction

| NCC Cutoff (#High Risk v.s. #Low Risk) | β(EC tau SUVr) in High Risk | β(EC tau SUVr) in Low Risk | p-value (β-EC tau SUVr in High Risk) | p-value (β-EC tau SUVr in Low Risk) |
| --- | --- | --- | --- | --- |
| 400 (18 v.s. 29) | 1.27 | 0.39 | **0.050** | 0.344 |
| 450 (21 v.s. 26) | 1.32 | 0.30 | **0.036** | 0.472 |
| **500 (24 v.s. 23)** | 1.33 | 0.24 | **0.022** | 0.576 |
| 550 (26 v.s. 21) | 1.02 | 0.36 | **0.038** | 0.455 |
| 600 (28 v.s. 19) | 0.87 | 0.60 | 0.064 | 0.241 |

Table S20. NCC Discrete Cutoff Subgroup Sensitivity in Original Logistic Regression Models (Conversion ~ tau SUVr in EC + MMSE + Age + Sex + EC/TIV Ratio) with Firth’s Bias Reduction

| NCC Cutoff (#High Risk v.s. #Low Risk) | β(EC tau SUVr) in High Risk | β(EC tau SUVr) in Low Risk | p-value (β-EC tau SUVr in High Risk) | p-value (β-EC tau SUVr in Low Risk) |
| --- | --- | --- | --- | --- |
| 400 (18 v.s. 29) | 5.40 | 0.23 | 0.052 | 0.640 |
| 450 (21 v.s. 26) | 5.84 | 0.24 | **0.048** | 0.628 |
| **500 (24 v.s. 23)** | 1.42 | 0.22 | **0.035** | 0.673 |
| 550 (26 v.s. 21) | 1.23 | 0.30 | **0.037** | 0.590 |
| 600 (28 v.s. 19) | 1.08 | 0.42 | **0.044** | 0.446 |
